# Digital Workflow with Open-Source CAD-CAM Software Aimed to Design a Customized 3D Laser-Printed Titanium Mesh for Guided Bone Regeneration

**DOI:** 10.3390/bioengineering12050436

**Published:** 2025-04-22

**Authors:** Claudio Cirrincione, Giulia Guarnieri, Annamaria Morelli

**Affiliations:** Department of Experimental and Clinical Medicine (DMSC), University of Florence, 50139 Florence, Italy; giulia.guarnieri@unifi.it (G.G.); a.morelli@unifi.it (A.M.)

**Keywords:** guided bone regeneration, CAD-CAM, titanium mesh, digital workflow, standard tessellation language, dental implant, pore width, stiffness, 3D printing

## Abstract

Guided bone regeneration (GBR) is a procedure used for the treatment of bone deficiencies. Computer-Aided Designed–Computer-Aided Manufacturing (CAD-CAM) allows us to design a titanium mesh (TM) for GBR directly on a 3D bone defect model (3DBM). The design and printing of TMs are often delegated to specialized 3D printing centers, thus preventing the surgeon from controlling surgical parameters such as the thickness, pore width, texture, and stiffness. Therefore, we have here proposed a personalized digital workflow for designing a TM. The 3DBM was uploaded to an open-source CAD-CAM software. Following a GBR simulation, a TM was designed as a Standard Tesselation Language (STL) file and 3D laser-printed. The TM was applied to a graft of 50/50% autologous/xenogenic bone, fixed with a bone screw, and covered with a dermal membrane. No TM exposure was observed during the healing phase. The regenerated bone volume was 970 cc, and pseudoperiosteum was class 1. At the 6-month reentry, a 4.1 × 10 standard dental implant with a primary stability of 40 N/cm was placed and after 3 months a zirconia crown screw-on implant was placed. This proposed digital workflow enabled us to successfully tackle this clinical case. However, further clinical investigations will be necessary to confirm the long-term benefits of this procedure.

## 1. Introduction

Guided bone regeneration (GBR) is a well-established oral surgical procedure aimed at reconstructing the alveolar bone defects that result from trauma, infection, or post-extraction resorption [1]. Alveolar ridge resorption is a common consequence of tooth loss due to the lack of functional stimuli on the underlying bone, leading to progressive atrophy, particularly on the buccal aspect. If untreated, this process compromises the possibility of placing a dental implant and requires a bone augmentation procedure [2]. GBR enables the restoration of lost alveolar dimensions, providing adequate bone volume for implant-supported rehabilitation, with favorable short-, medium-, and long-term results for the stability of peri-impant bone levels [3]. The GBR technique involves the placement of a bone graft—either autogenous, xenogenic, or alloplastic—beneath a barrier membrane or mesh to prevent soft tissue ingrowth and facilitate osteogenesis [4]. Of the different types of membranes available, non-resorbable options, such as titanium-reinforced polytetrafluoroethylene (PTFE) membranes and titanium meshes (TMs), are widely used [5]. TMs, in particular, have gained popularity due to their superior biocompatibility, mechanical stability, and ability to maintain space, thereby providing the required “tenting effect” for bone regeneration. The mechanical properties of TMs allow for predictable outcomes, with their reported success rates in bone augmentation ranging from 87% to 98%, depending on the defect’s size, biomaterial selection, and the surgical technique used [6]. Despite their advantages, the intraoperative adaptation of TMs remains a significant challenge. Manual shaping and contouring are time-consuming, technique-sensitive, and highly dependent on the operator’s experience, increasing the risk of misfit, exposure, and mucosal dehiscence [7,8,9,10]. In this context, the introduction of Computer-Aided Design and Computer-Aided Manufacturing (CAD-CAM) technology has revolutionized the field of GBR. CAD-CAM enables the precise preoperative design of custom-made meshes with optimized geometry, adequate pore dimensions to minimize soft tissue complications, and controlled stiffness to balance mechanical stability with biological integration. Conventional CAD-CAM workflows rely on third-party digital centers that process Cone Beam Computed Tomography (CBCT) scans in a Digital Imaging and Communications in Medicine (DICOM) format, generate three-dimensional virtual stereolitographic models using a Standard Tesselation Language (STL), and fabricate the final mesh using 3D printing technology, such as titanium laser sintering/melting [11,12,13]. While effective, this approach limits the surgeon’s direct control over key mesh design parameters, such as its thickness, pore distribution, and flexibility [14]. Moreover, the high costs associated with proprietary software and external manufacturing services restrict accessibility for many clinicians [15]. TMs have biomechanical benefits and drawbacks compared to other GBR barriers, such as the collagen membranes or PTFE barriers. While PTFE membranes provide a protective enclosure with minimal risk of soft tissue penetration, they lack the rigidity required for significant volume augmentation. Similarly, collagen membranes exhibit superior biocompatibility but undergo resorption, making them unsuitable for large or vertical bone defects. A direct comparison of clinical outcomes suggests that custom CAD-CAM TMs achieve higher volumetric stability than PTFE and collagen membranes, particularly in vertical augmentation procedures [16].

To address these limitations, this study presents a novel digital workflow that allows surgeons to independently design customized TMs using an open-source CAD software platform [17]. This approach enhances flexibility, reduces costs, and improves surgical planning by allowing real-time adjustments to the mesh’s thickness, porosity, and anatomical fit. This study also includes a step-by-step demonstration of the process, from CBCT image acquisition to mesh design and surgical application, highlighting its advantages over conventional methods. By integrating CAD-CAM technology with an open-source framework, this study aims to provide a reproducible and accessible solution for clinicians seeking to optimize GBR procedures.

## 2. Materials and Methods

**Figure 1:**
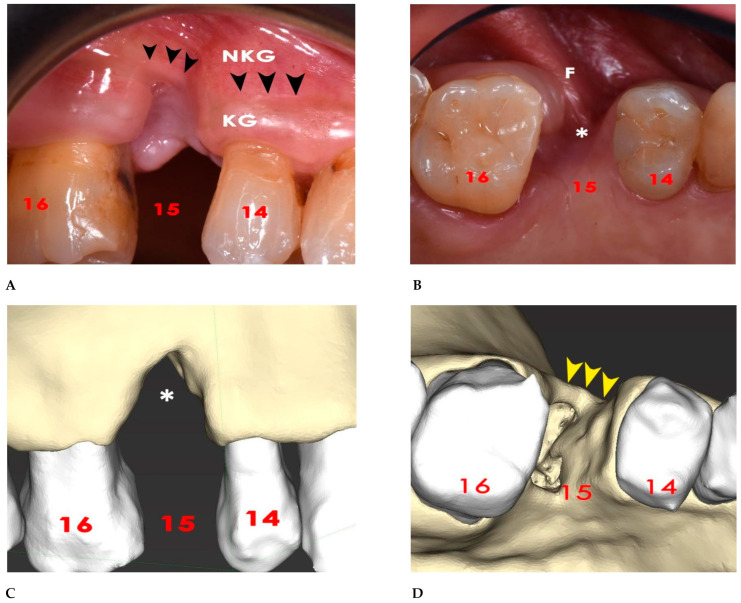
(**A**) At the site of tooth 15, keratinized gingiva (KG) was lost up to the junction with the non-keratinized gingiva (NKG). Arrows indicate the junction between the KG and NKG. (**B**) A frenulum (F) was inserted in the apical part of the gingival defect (*). (**C**) The 3D bone defect model resulting from the DICOM data of the baseline CBCT has a frontal “V”-shaped vertical defect with loosening of the entire alveolar process (*). (**D**) An occlusal view showed the horizontal extent of the bone alveolar defect (arrows).

This article followed the STROBE (Strengthening the Reporting of Observational Studies in Epidemiology) guidelines and built upon previous research on digital workflows, GBR, customized titanium meshes, and implant-supported bone regeneration. The treatment was conducted according to the principles of the Helsinki Declaration of 1975 for biomedical research involving human subjects, which were revised in 2013. The patient was informed of the nature of the study, the benefits, risks, and possible alternative treatments, and also gave their written consent for the use of clinical images. The clinical case we refer to was a 60-year-old patient who complained of esthetic and functional problems that arose following a previous dental extraction. At the first oral examination, there was visible a buccal and palatal gingival defect with a vertical component at the site of tooth 15. Attached keratinized gingiva was lost near the border of the alveolar non-keratinized mucosa (Figure 1A), with a frenulum inserted in the apical portion of the defect (Figure 1B). A first baseline CBCT was taken using an high-resolution tool (Promax 3D Max;Planmeca, Finland) and the following scanning parameters: FOV 100 × 55 mm, 94 Kv, 10 mA, 8 s pulsed emission, and 200 µm resolution. The CBCT showed a “V”-shaped combined vertical and horizontal bone defect better seen with a 3D representation (Figure 1C,D). Given the difficulty in placing an implant in an ideal position, a staged GBR was planned in which a customized TM would be designed and used as space-making device and a collagen membrane would be applied as barrier device in order to optimize bone regeneration [18].

### 2.1. Surgical Simulation

**Figure 2:**
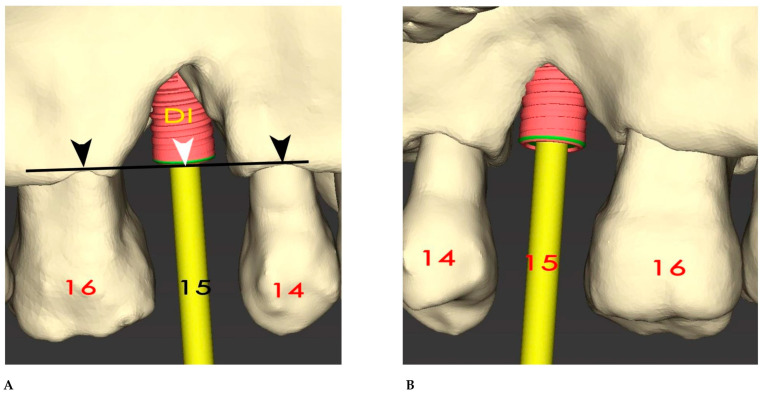
(**A**) Virtual 3D insertion of a regular 10 mm dental implant (DI) at the site of tooth 15. The dental implant’s coronal border (white arrow) was aligned with most of the coronal bone level of the adjacent teeth, 16 and 14 (black arrows). Nevertheless, almost all of the implant’s body was outside the residual bone. (**B**) The palatal view showed a similar situation to the vestibular view.

An initial digital intraoral impression was taken (Medit i500, Medit; MEDIT Co., Seoul, Republic of Korea). The resulting Standard Tesselation Language (STL) files were merged with the DICOM data derived from the CBCT using surgical diagnosis and planning software (coDiagnostiX, Dental Wings, version 10.8, USA). The segmentation accuracy of the DICOM files was significantly improved thanks to the on-demand Artificial Intelligence function provided by this software. At site 15, a regular-diameter dental implant was virtually inserted (4.1 × 10 mm Bone Level Tapered, Straumann, Basel, Switzerland). The surface of the implant was almost totally outside of the residual bone, and thus a staged GBR procedure was mandatory (Figure 2A,B).

### 2.2. GBR Simulation and Mesh Design

**Figure 3:**
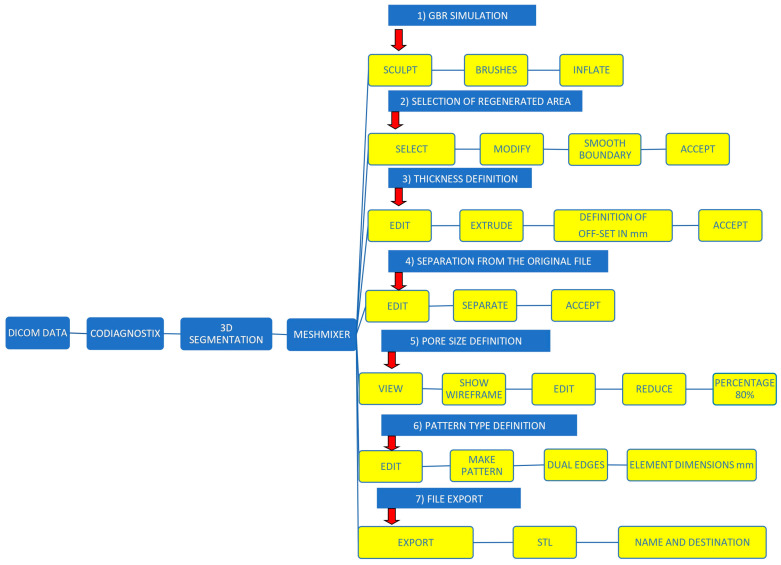
Personalized digital workflow for designing a mesh for GBR.

**Figure 4:**
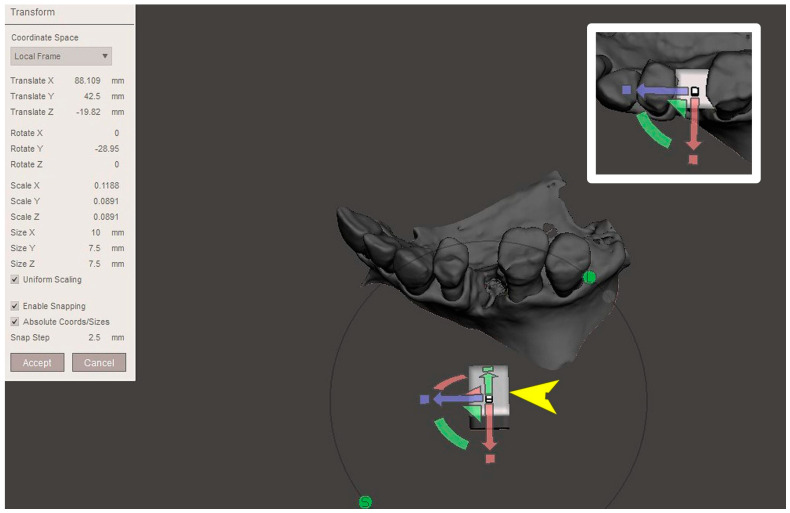
View of the open-source CAD-CAM software’s desktop. The yellow arrow shows the cubic STL file that is to be transformed and matched with the virtual bone model. The upper right panel shows the integration of the transformed cubic STL file with the virtual bone defect model.

**Figure 5:**
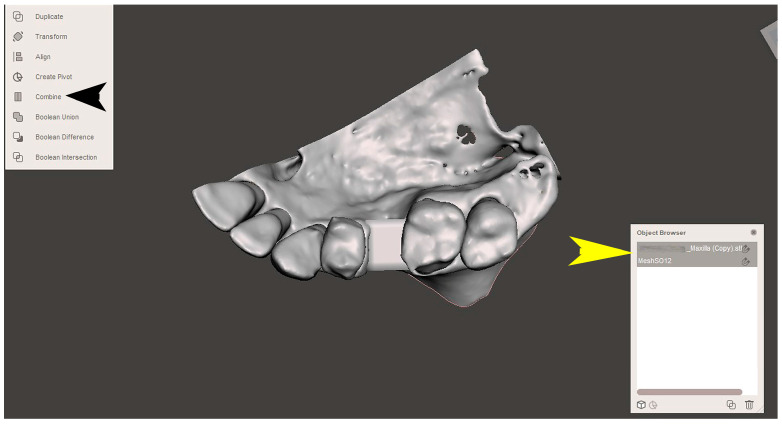
The matching of the two files (“combine” command, black arrow) results in a single STL file (the yellow arrow indicates the object browser).

**Figure 6:**
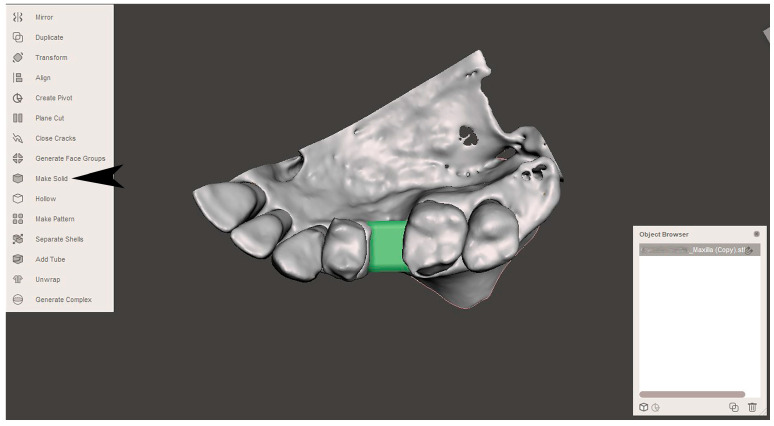
The resulting file needs to be transformed into a solid file (the black arrow indicates the “Make solid” command).

**Figure 7:**
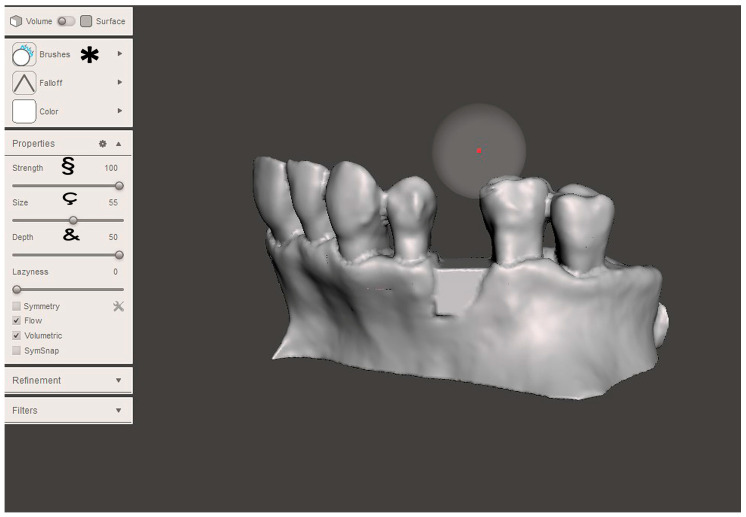
With some commands (step 1, “Brush”, see *), it was possible to smooth the file’s surface. Other commands enabled us to strengthen (“Strength”, see §) the smoothing activity, while others allowed us to expand (“Size”, see ç) or deepen (“Depth”, see &) the working area.

**Figure 8:**
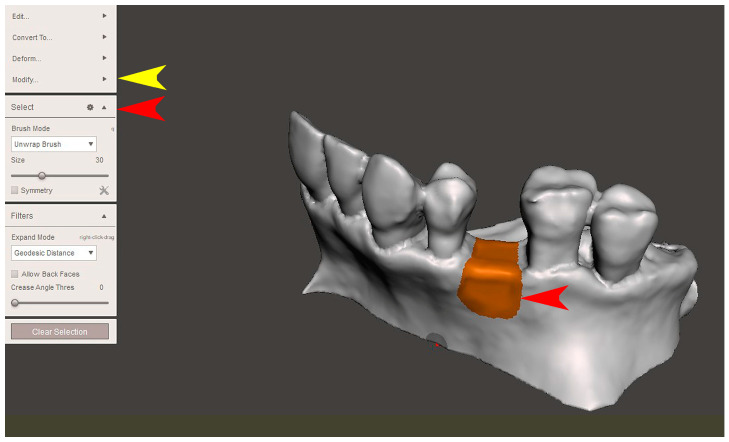
In step 2, the surface above the defect was selected (red arrows, “Select” command) and then the borders were smoothed (yellow arrow, “Modify” and “Smooth boundary” commands).

**Figure 9:**
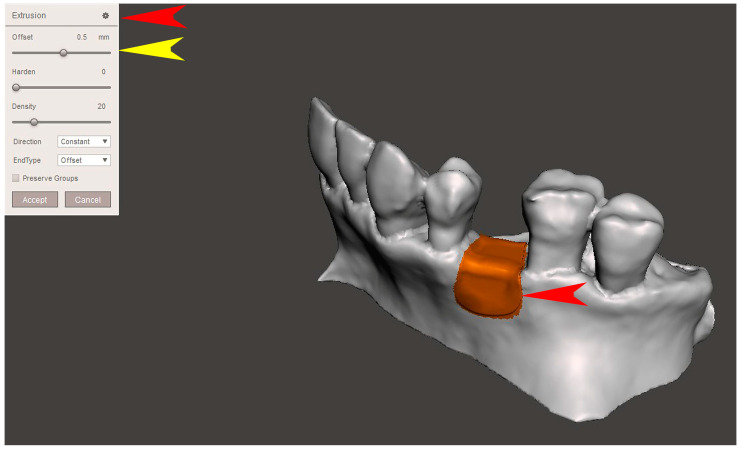
In step 3, the selected surface was extruded (“Extrusion”, red arrows) and then its thickness was defined (“Off-set”, yellow arrow).

**Figure 10:**
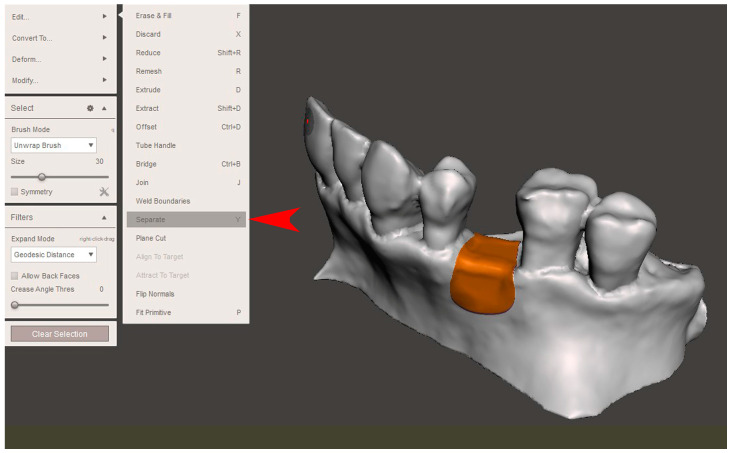
In step 4, the mesh was separated from the virtual bone defect model (“Separate”, red arrow).

**Figure 11:**
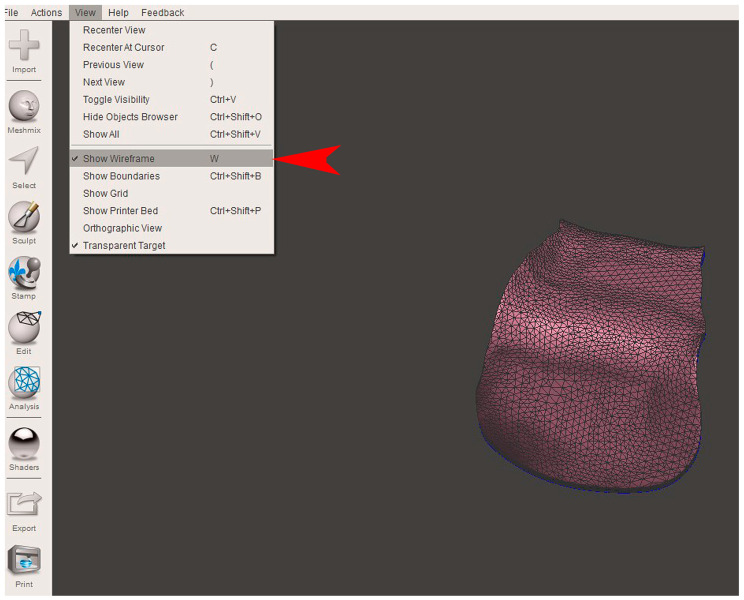
In step 5, in order to modify the mesh pore width, it was necessary to visualize the triangle composition of the STL file (“Show wireframe”, red arrow).

**Figure 12:**
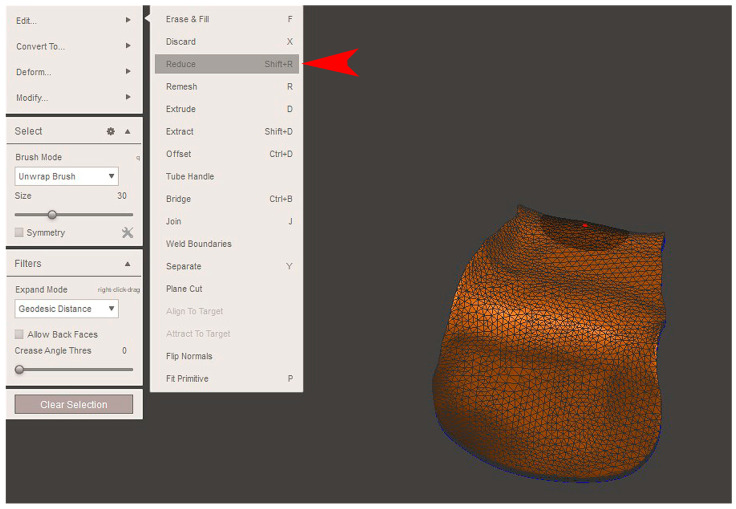
A reduction in the number of triangles by the desired percentage was achieved (“Reduce”, red arrow). The width of the pore will affect the stiffness of the titanium mesh and the periosteal perfusion of blood nutrients inside the bone graft.

**Figure 13:**
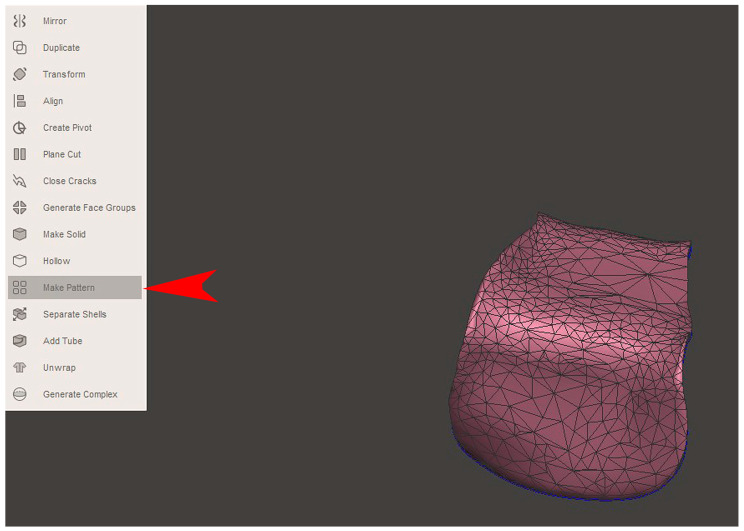
In step 6, an adequate texture for the mesh was defined (“Make Pattern”, red arrow).

**Figure 14:**
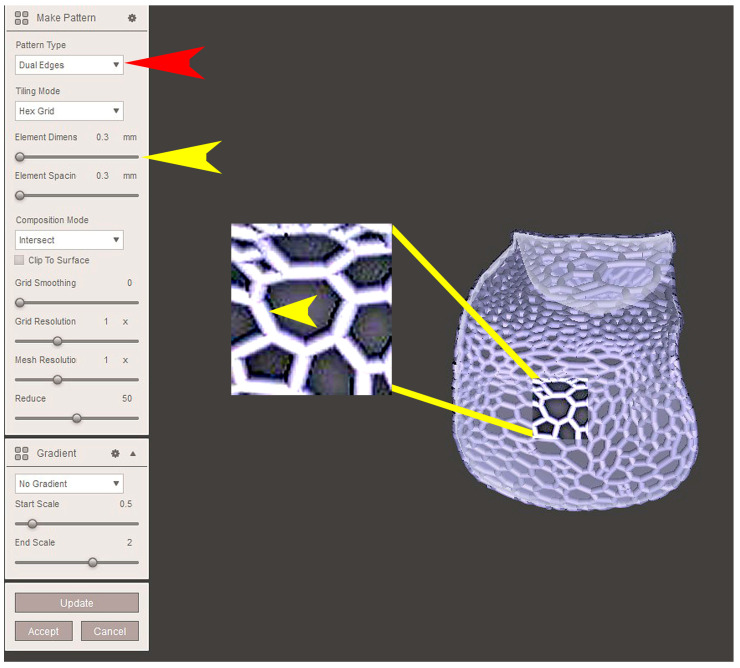
Once the texture was selected (“Pattern type”, “Dual Edges”, red arrow), the thickness of the mesh elements was defined as well (“Element dimension”, yellow arrows, see also the magnification of a section for more detail). The thickness of the mesh elements will affect the stiffness of the titanium mesh.

**Figure 15:**
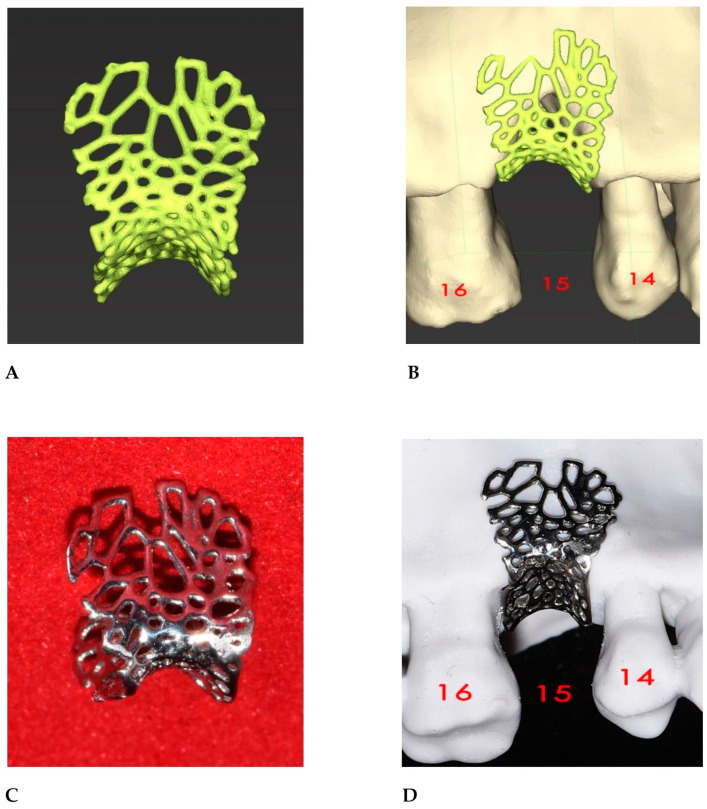
(**A**) A 3D representation of the mesh. (**B**) In step 7, the mesh was uploaded into the diagnosis and planning software to verify its fit with the virtual bone defect model. (**C**) The 3D laser-printed titanium mesh. The more polished the mesh, the less difficult it will be to remove during the second surgical step. (**D**) Titanium mesh placed on a 3D-printed model of the bone defect.

The STL file of the virtual bone defect model showing the initial alveolar bone atrophy was imported into open-source CAD-CAM software (OSCS, Meshmixer, Version 3.5.474, Autodesk, San Francisco, CA, USA). Here, following a personalized digital workflow (Figure 3), bone regeneration and mesh design procedures were both simulated. Using some sculpting commands, the bone defect was completely filled, reproducing the bone level and the shape of the adjacent intact alveolar process (step 1). In certain clinical cases, before step 1, some free STL files available on OSCS that match with the original file can be used. For instance, a cube STL file may be adapted (Figure 4), matched with the bone atrophy model (Figure 5), made into a solid file (Figure 6), and then sculpted with the sculpting commands (Figure 7). To develop the mesh, the regenerated zone was selected (Figure 8, step 2) and the thickness of the mesh (Figure 9, step 3); its pore width; and its texture, in this case a Voronoi texture, were adjusted (Figure 10, Figure 11, Figure 12, Figure 13 and Figure 14, step 4 to 6). The thickness of the mesh was set to 0.5 mm, while the pore width had a medium size of 0.3 mm. The resulting STL file (Figure 15A) was uploaded to the diagnosis and planning software (step 7) to verify its union with the bone defect (Figure 15B). Therefore, the mesh was 3D printed in titanium alloy (Ti6Al4V) using a laser melting procedure, subjected to a stress relief firing treatment in Argon at 850 °C for 120 min, sand blasted with aluminum oxide (200 µ at 3 bar), and hand polished on its upper and lower sides (Figure 15C, CADdent GmbH, Max-Jozef-Metzger-Str.6, Augsburg, Germany). A replica of the bone defect was 3D printed (Ackuretta Tech, Taipei City, Taiwan, China) to verify the accuracy of the fit (Figure 15D), and then the mesh was autoclave-sterilized.

### 2.3. Surgical and Prosthetic Procedures

**Figure 16:**
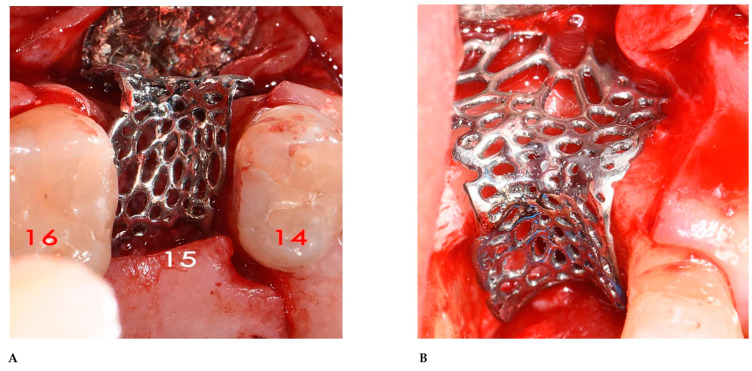
(**A**) Initial fitting of the titanium mesh over the bone defect, occlusal view. (**B**) Final positioning of the titanium mesh over the bone defect.

**Figure 17:**
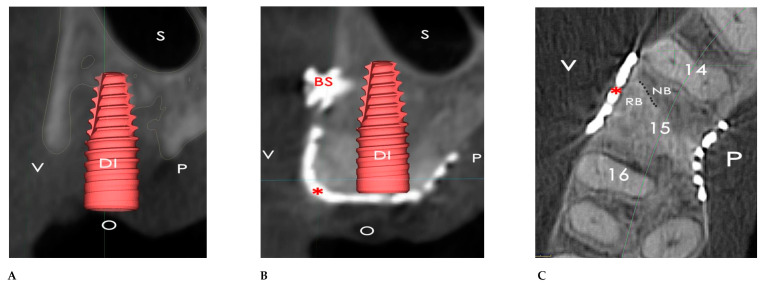
(**A**) Vestibule–palatal section of the baseline CBCT. The dental implant (DI) was almost uncovered by bone. (**B**) Vestibule–palatal section of 6-month secondary CBCT. A regular-size 10 mm dental implant (DI) was inserted in place of tooth 15. The titanium mesh (*) was integrated with the new-formed alveolar bone, without any black zone between the mesh and the regenerated bone. BS: bone screw; V: vestibulum; P: palate; O: occlusal; S: maxillary sinus. (**C**) Transversal section of the 6-month follow-up CBCT showed the integration between the native alveolar bone (NB) and the regenerated bone (RB), which is outlined with a dotted line. *: titanium mesh; V: vestibulum; P: palate.

**Figure 18:**
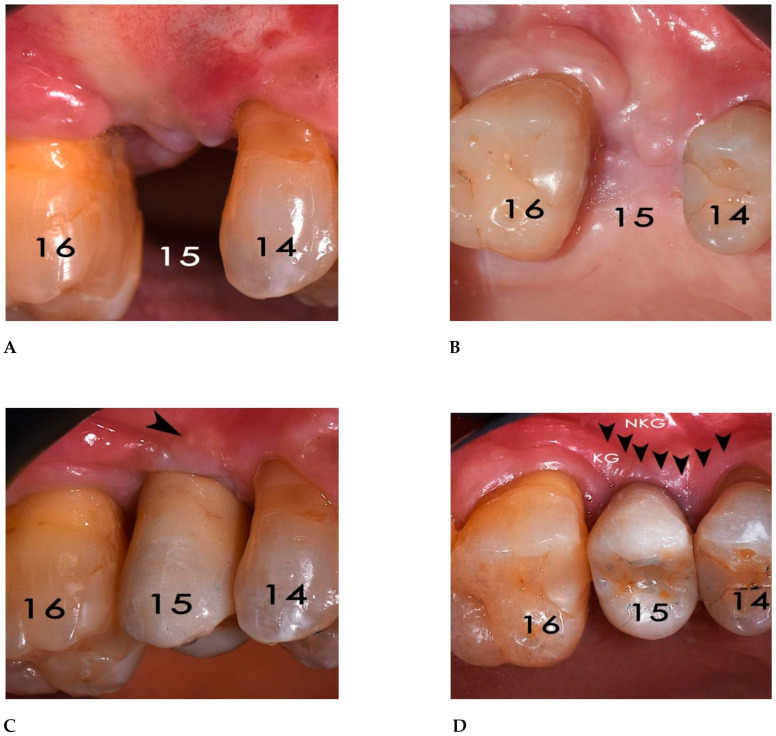
(**A**) Frontal view of the regenerated zone at 6-month follow-up. (**B**) Occlusal view shows color and texture uniformity of the gingival tissue without any sign of mesh dehiscence. (**C**) Delivery of the crown. Some granules of xenogenic bone were visible beneath the gingival tissue (arrow). (**D**) Occlusal view shows the restoration of the keratinized gingiva (KG). Arrows: junction between keratinized and non-keratinized gingiva (NKG).

Following local anesthetic infiltration (4% Articaine, Septanest with adrenaline 1/100,000, Septodont, Saint-Maur-des-fossés, France), a full-thickness mucoperiosteal flap was elevated with a mid-crestal and two vertical incisions, one on the disto-buccal side and the other on the disto-palatal side of tooth 14. A second full-thickness mucoperiosteal flap was elevated in the tuberosity zone. Here, a bone graft was harvested as a block with the help of a piezoelectric device (SONICflex bone^®^ 2003L, KaVo Dental, Biberach, Germany) and subsequently ground and fragmented using a bone mill (Bone mill, Carl Martin GmbH, Solingen, Germany). Using the patient’s blood, the graft was admixed with small particules of xenogenic bone substitute (BioOss; Geistlich Pharma AG, Wolhusen, Switzerland) in a 50/50 ratio and used to fill the bone defect. The titanium mesh was applied to the bone defect to cover the graft (Figure 16A,B), fixed with a bone screw (1.6 × 8 titanium screw, Storz am Mark, Emmingen-Liptingen, Germany) on the buccal side, and covered with a xenogeneic acellular dermal matrix (Mucoderm^®^, Botiss Biomaterials, Zossen, Germany) to reduce the risk of TM exposure, as outlined by Cucchi et al. [18]. A horizontal periosteal incision was made to achieve buccal flap advancement and the primary closure of the wound, and then interrupted sutures (3.0 Perma-Hand silk, Ethicon, Somerville, NJ, USA) were utilized to close the two surgical sites. Oral antibiotics, amoxicillin and clavulanic acid, at 2 g/die for 6 days (Augmentin, GSK, London, UK), and a nonsteroidal anti-inflammatory drug, at 1 g/die for 2 days (Aulin, Angelini Pharma SPA, Rome, Italy), were prescribed. The patient was instructed to rinse with 0.2% chlorhexidine twice a day for 10 days and apply ice packs to the surgical site for 48 h. The sutures were removed 15 days after the surgery. After 6 months of bone healing, a second CBCT with the same parameters as that of the baseline was taken (Figure 17A–C) to compare the result with the original clinical view (Figure 18A,B). A full-thickness mucoperiosteal flap was elevated and the bone screw and the mesh were removed. Then, a regular-size implant was placed (4.1 × 10 mm, Bone Level Tapered; Straumann, Switzerland). After a healing period of 3 months, a split-thickness apically repositioned flap was raised to reduce the muscular traction of the frenulum. Fifteen days later, a new digital impression was taken and a zirconia crown screw-on implant was placed (Figure 18C,D).

## 3. Results

The OSCS used in this work allowed us to design and realize a very precise mesh for GBR. This resulted in reducing the surgical time to 40 min and the absence of any surgical or technical complications, except for a small mucosal dehiscence at the time of suture removal, without TM exposure during the months that followed.

As analyzed in the OSCS, the planned bone volume was 1.024 cc, while the regenerated bone volume was 970 cc., with the ratio between the planned bone volume and regenerated bone volume calculated to be 94.7%.

By the time of the re-entry surgery, the bone defect was completely filled with newly formed bone of a medium bone density and pseudoperiosteum class 1 [19]. The vertical bone gain was 3.5 mm, the horizontal bone gain was 5.6 mm, and the regenerated bone volume was 970 cc. The appearance and consistency of the regenerated bone was similar to the native tissue, with no presence of any fibrous tissue. The insertion of the implant reached 40 Ncm of torque.

## 4. Discussion

This clinical case has shown that a GBR and implant–prosthetic procedure can be successfully realized with a personalized digital workflow. To our knowledge, this is the first work explaining in detail the steps required to design a 3D mesh for GBR using a staged approach. Moreover, designing the mesh with the OSCS has allowed us to realize a precise digital structure, with the 3D-printed TM easily adapted to the bone defect.

Before the introduction of CAD-CAM technology, achieving perfect adaptation of a titanium mesh to the defect was a time-consuming procedure that depended heavily on the manual skills of the surgeon. Inadequate adaptation often led to surgical failures, including wound dehiscence and bone graft loss [20,21,22,23]. The digital design of the titanium mesh has significantly reduced the occurrence of these complications, although it has not entirely eliminated them, as observed in our clinical case; the mechanical matching between the rigidity of the titanium mesh and the oral mucosa may be one of the reasons for mucosal cracking after surgery. However, several other factors may contribute to wound dehiscence, including the periosteal incision and accidental occlusal load, which may impair capillary perfusion and increase the risk of soft tissue complications [24,25,26]. The issue of wound dehiscence and mesh exposure is crucial because it is significantly related to the risk of mesh infection and consequent graft loss, which may affect the results of the bone augmentation [27,28]. Furthermore, the difference in stiffness between the oral mucosa and the titanium mesh has also been reported as a factor contributing to wound dehiscence. However, covering the mesh with a dermal membrane can partially mitigate this issue, promoting faster epithelialization and mucosal thickening [29,30,31]. Future advancements involving biodegradable 3D-printed polymeric meshes may further reduce this phenomenon [32,33]. Despite these promising outcomes, this study has several limitations. First, no secondary stability measurements of the dental implant were performed. Implant stability is a crucial parameter for assessing treatment success, and future studies should incorporate resonance frequency analysis (RFA) values to better evaluate implant integration [34,35]. Additionally, a comparison with baseline values or previous data from the literature is needed to contextualize these findings. Second, while this study provides a detailed protocol for the CAD-CAM design of a 3D-printed TM, no systematic analysis of potential inaccuracies in the CAD-CAM workflow was conducted. Factors such as STL file resolution, digital segmentation errors, and printing accuracy could impact the final fit and clinical performance of the customized TM. Studies evaluating the dimensional precision of CAD-CAM titanium meshes have highlighted potential deviations between the digital design and the fabricated mesh, which could affect clinical outcomes [36,37]. Third, this study was limited to a single clinical case without a control group. While our findings align with prior research that has demonstrated the advantages of customizing CAD-CAM titanium meshes using an OSCS for GBR, they should be validated through larger-scale clinical trials [38]. Nevertheless, our results are consistent with previous research, supporting the use of customized CAD-CAM titanium meshes for alveolar ridge augmentation. Several studies have reported high success rates for GBR procedures using these meshes, with bone gains ranging from 4.3 mm in width to 4.11 mm in height and implant survival rates exceeding 95%. Moreover, customized CAD-CAM TMs have been associated with improved volumetric stability compared to non-resorbable PTFE membranes or collagen barriers, particularly in vertical augmentation procedures [39,40].

Although whether the software encountered technical difficulties during the design process and whether these problems may affect the design quality and surgical results of the titanium mesh remain to be seen, the integration of OSCS into GBR and implant surgery has introduced a cost-effective, customizable, and transparent alternative to the proprietary systems. The ability to design customized 3D-printed TMs with full control over the digital workflow enhances surgical efficiency and precision.

Unlike commercial software, open-source solutions eliminate licensing fees, making advanced GBR techniques more accessible. Additionally, open-source platforms allow for fully customizable mesh designs, including optimizing parameters like thickness, porosity, and stiffness for patient-specific needs. This flexibility minimizes surgical complications such as wound dehiscence and graft resorption.

Traditional CAD-CAM workflows rely on third-party digital centers, limiting the surgeon’s direct involvement in mesh adaptation. Open-source software allows for real-time adjustments to STL files, improving precision and reducing dependency on external providers. Additionally, these tools support multiple file formats, ensuring compatibility with various 3D printing technologies.

One key advantage of open-source platforms is the full visibility of their design algorithms, which enhances their scientific validation and allows researchers to refine GBR techniques. Open-source software fosters collaborative innovation, enables AI-driven design optimization, and improves the accuracy of surgical planning.

Despite these benefits, open-source software requires technical expertise, and potential errors in STL segmentation and mesh adaptation must be addressed to ensure accuracy. Future advancements in biodegradable 3D-printed meshes and AI-assisted CAD-CAM design may further enhance GBR outcomes.

## 5. Conclusions

In conclusion, this study highlights the advantages of a personalized digital workflow that uses open-source CAD-CAM software for designing and fabricating a customized 3D-printed mesh for GBR.

While this clinical case provides a detailed step-by-step protocol, further clinical investigations, including randomized controlled trials and comparisons with other CAD-CAM software, will be necessary to confirm the long-term clinical benefits of this new digital workflow in GBR.

## Data Availability

Data are available upon reasonable request.

## Abbreviations

The following abbreviations are used in this manuscript:

GBR	Guided Bone Regeneration
TM	Titanium Mesh
CAD-CAM	Computer-Aided Design–Computer-Aided Manufacturing
STL	Standard Tesselation Language
DICOM	Digital Imaging and Communications in Medicine
CBCT	Cone Beam Computed Tomography
OSCS	Open-Source CAD-CAM Software

## References

[1] Mateo-Sidrón Antón M.C., Pérez-González F., Meniz-García C. (2024). Titanium mesh for guided bone regeneration: A systematic review. Br. J. Oral Maxillofac. Surg..

[2] Urban I.A., Montero E., Monje A., Sanz-Sánchez I. (2019). Effectiveness of vertical ridge augmentation interventions: A systematic review and meta-analysis. J. Clin. Periodontol..

[3] Cucchi A., Maiani F., Franceschi D., Sassano M., Fiorino A., Urban I.A., Corinaldesi G. (2024). The influence of vertical ridge augmentation techniques on peri-implant bone loss: A systematic review and meta-analysis. Clin. Implant Dent. Relat. Res..

[4] Roccuzzo M., Ramieri G., Spada M.C., Bianchi S.D., Berrone S. (2004). Vertical alveolar ridge augmentation by means of a titanium mesh and autogenous bone grafts. Clin. Oral Implants Res..

[5] Alotaibi F.F., Rocchietta I., Buti J., D’Aiuto F. (2023). Comparative evidence of different surgical techniques for the management of vertical alveolar ridge defects in terms of complications and efficacy: A systematic review and network meta-analysis. J. Clin. Periodontol..

[6] Sabri H., Heck T., Manouchehri N., Alhachache S., Calatrava J., Misch C.M., Wang H.L. (2024). Bone augmentation using titanium mesh: A systematic review and meta-analysis. Int. J. Oral Implantol..

[7] Lizio G., Pellegrino G., Corinaldesi G., Ferri A., Marchetti C., Felice P. (2022). Guided bone regeneration using titanium mesh to augment 3-dimensional alveolar defects prior to implant placement. A pilot study. Clin. Oral Implants Res..

[8] Cirrincione C., Ricci M., Guarnieri G., Morelli A., Ottanelli G., Piccarreta E. (2024). Excessive stiffness of meshes for oral guided bone regeneration may cause mucosal dehiscence: An in-vitro comparative loading study between titanium alloy and polycaprolactone. Ital. J. Anat. Embryol..

[9] Cucchi A., Vignudelli E., Napolitano A., Marchetti C., Corinaldesi G. (2017). Evaluation of complication rates and vertical bone gain after guided bone regeneration with non-resorbable membranes versus titanium meshes and resorbable membranes. A randomized clinical trial. Clin. Implant Dent. Relat. Res..

[10] Cucchi A., Bettini S., Ghensi P., Fiorino A., Corinaldesi G. (2023). Vertical ridge augmentation with Ti-reinforced dense polytetrafluoroethylene (d-PTFE) membranes or Ti-meshes and collagen membranes: 3-year results of a randomized clinical trial. Clin. Implant Dent. Relat. Res..

[11] Ciocca L., Ragazzini S., Fantini M., Corinaldesi G., Scotti R. (2015). Work flow for the prosthetic rehabilitation of atrophic patients with a minimal-intervention CAD/CAM approach. J. Prosthet. Dent..

[12] Seiler M., Kämmerer P.W., Peetz M., Hartmann A.G. (2018). Customized Titanium Lattice Structure in Three-Dimensional Alveolar Defect: An Initial Case Letter. J. Oral Implantol..

[13] Cucchi A., Bianchi A., Calamai P., Rinaldi L., Mangano F., Vignudelli E., Corinaldesi G. (2020). Clinical and volumetric outcomes after vertical ridge augmentation using computer-aided-design/computer-aided manufacturing (CAD/CAM) customized titanium meshes: A pilot study. BMC Oral Health.

[14] Chiapasco M., Casentini P., Tommasato G., Dellavia C., Del Fabbro M. (2021). Customized CAD/CAM titanium meshes for the guided bone regeneration of severe alveolar ridge defects: Preliminary results of a retrospective clinical study in humans. Clin. Oral Implants Res..

[15] Tommasato G., Piano S., Casentini P., De Stavola L., Chiapasco M. (2024). Digital planning and bone regenerative technologies: A narrative review. Clin. Oral Implants Res..

[16] Cucchi A., Bettini S., Tedeschi L., Urban I., Franceschi D., Fiorino A., Corinaldesi G. (2024). Complication, vertical bone gain, volumetric changes after vertical ridge augmentation using customized reinforced PTFE mesh or Ti-mesh. A non-inferiority randomized clinical trial. Clin. Oral Implants Res..

[17] Cirrincione C. Finite Element Analysis comparison between a polycaprolactone and a Ti6Al4V alloy meshs for bone reconstruction, designed with open-source CAD software: Importance of the mesh thickness and pore width. Proceedings of the ITI World Symposium Singapore.

[18] Cucchi A., Vignudelli E., Franceschi D., Randellini E., Lizio G., Fiorino A., Corinaldesi G. (2021). Vertical and horizontal ridge augmentation using customized CAD/CAM titanium mesh with versus without resorbable membranes. A randomized clinical trial. Clin. Oral Implants Res..

[19] Cucchi A., Sartori M., Aldini N.N., Vignudelli E., Corinaldesi G. (2019). A Proposal of Pseudo-periosteum Classification After GBR by Means of Titanium-Reinforced d-PTFE Membranes or Titanium Meshes Plus Cross-Linked Collagen Membranes. Int. J. Periodontics Restor. Dent..

[20] Uehara S., Kurita H., Shimane T., Sakai H., Kamata T., Teramoto Y., Yamada S. (2015). Predictability of staged localized alveolar ridge augmentation using a micro titanium mesh. Oral Maxillofac. Surg..

[21] Sumida T., Otawa N., Kamata Y.U., Kamakura S., Mtsushita T., Kitagaki H., Mori S., Sasaki K., Fujibayashi S., Takemoto M. (2015). Custom-made titanium devices as membranes for bone augmentation in implant treatment: Clinical application and the comparison with conventional titanium mesh. J. Craniomaxillofac. Surg..

[22] Lee S.Y., Choi S.H., Lee D.W. (2024). Vertical Ridge Augmentation with Customized Titanium Mesh Using a 3D-Printing Model: A Prospective Study in Humans. Int. J. Oral Maxillofac. Implants.

[23] Lorusso F., Gehrke S.A., Alla I., Tari S.R., Scarano A. (2025). The Early Exposure Rate and Vertical Bone Gain of Titanium Mesh for Maxillary Bone Regeneration: A Systematic Review and Meta-Analysis. Dent. J..

[24] Cirrincione C., Ottanelli G. (2024). Three-dimensionally printed polycaprolactone shows more physiological stiffness compared with titanium alloy. Proceedings of the 1st International Online Conference on Functional Biomaterials.

[25] Park S.H., Wang H.L. (2007). Clinical significance of incision location on guided bone regeneration: Human study. J. Periodontol..

[26] Wang C.X., Rong Q.G., Zhu N., Ma T., Zhang Y., Lin Y. (2023). Finite element analysis of stress in oral mucosa and titanium mesh interface. BMC Oral Health.

[27] Liu C., Li J., Zhang S., Xiao H., Wang Y., Zhang J. (2024). Assessment of the application of a novel three-dimension printing individualized titanium mesh in alveolar bone augmentation: A retrospective study. Clin. Implant Dent. Relat. Res..

[28] Lizio G., Mazzone N., Corinaldesi G., Marchetti C. (2016). Reconstruction of Extended and Morphologically Varied Alveolar Ridge Defects with the Titanium Mesh Technique: Clinical and Dental Implants Outcomes. Int. J. Periodontics Restor. Dent..

[29] Ronda M., Desantis V., Bruno D., Veneriano L., Elli C., Pispero A. (2024). New Generation Customized Titanium Meshes for the Guided Bone Regeneration of Severe Alveolar Ridge Defects: Preliminary Results of a Retrospective Case Series. Int. J. Periodontics Restor. Dent..

[30] Felice P., Pistilli R., Pellegrino G., Bonifazi L., Tayeb S., Simion M., Barausse C. (2024). A randomised controlled trial comparing the effectiveness of guided bone regeneration with polytetrafluoroethylene titanium-reinforced membranes, CAD/CAM semi-occlusive titanium meshes and CAD/CAM occlusive titanium foils in partially atrophic arches. Int. J. Oral Implantol..

[31] Vilanova-Corrales P., Demiquels-Punzano E., Caballé-Serrano J., Hernández-Alfaro F., Delgado J.Á., Pérez R.A., Gil J., Delgado L.M. (2024). Biodegradable and reinforced membranes based on polycaprolactone and collagen for guided bone regeneration. Mater. Today Commun..

[32] Bartnikowski M., Vaquette C., Ivanovski S. (2020). Workflow for highly porous resorbable custom 3D printed scaffolds using medical grade polymer for large volume alveolar bone regeneration. Clin. Oral Implants Res..

[33] Ivanovski S., Staples R., Arora H., Vaquette C., Alayan J. (2024). Alveolar bone regeneration using a 3D-printed patient-specific resorbable scaffold for dental implant placement: A case report. Clin. Oral Implants Res..

[34] Tanaka K., Sailer I., Iwama R., Yamauchi K., Nogami S., Yoda N., Takahashi T. (2018). Relationship between cortical bone thickness and implant stability at the time of surgery and secondary stability after osseointegration measured using resonance frequency analysis. J. Periodontal Implants Sci..

[35] Brouwers J.E.I.G., Buis S., de Groot P.G., de Laat B., Remijn J.A. (2021). Resonance frequency analysis with two different devices after conventional implant placement with ridge preservation: A prospective pilot cohort study. Clin. Implant Dent. Relat. Res..

[36] Chen D., Zheng L., Wang C., Huang Y., Huang H., Apicella A., Hu G., Wang L., Fan Y. (2023). Evaluation of surgical placement accuracy of customized CAD/CAM titanium mesh using screws-position-guided template: A retrospective comparative study. Clin. Implant Dent. Relat. Res..

[37] Li L., Wang C., Li X., Fu G., Chen D., Huang Y. (2021). Research on the dimensional accuracy of customized bone augmentation combined with 3D-printing individualized titanium mesh: A retrospective case series study. Clin. Implant Dent. Relat. Res..

[38] Urban I., Montero E., Sanz-Sánchez I., Palombo D., Monje A., Tommasato G., Chiapasco M. (2023). Minimal invasiveness in vertical ridge augmentation. Periodontology 2000.

[39] Simion M., Pistilli R., Vignudelli E., Pellegrino G., Barausse C., Bonifazi L., Roccoli L., Iezzi G., Felice P. (2023). Semi-occlusive CAD/CAM titanium mesh for guided bone regeneration: Preliminary clinical and histological results. Int. J. Oral Implantol..

[40] Hartmann A., Hildebrandt H., Younan Z., Al-Nawas B., Kämmerer P.W. (2022). Long-term results in three-dimensional, complex bone augmentation procedures with customized titanium meshes. Clin. Oral Implants Res..

